# Effectiveness of prophylactic antibacterial drugs for patients with liver cirrhosis and upper gastrointestinal bleeding: a systematic review and meta-analysis

**DOI:** 10.3389/fphar.2024.1324848

**Published:** 2024-03-14

**Authors:** Zhuo Wang, Han-Shuo Hu, Li-Mei Zhao, Yu Li, Xiao-Dong Liu

**Affiliations:** ^1^ Department of Pharmacy, Shengjing Hospital of China Medical University, Shenyang, China; ^2^ Department of the Second Clinical Pharmacy, School of Pharmacy, China Medical University, Shenyang, China; ^3^ Department of Pulmonary and Critical Care Medicine, Shengjing Hospital of China Medical University, Shenyang, China

**Keywords:** liver cirrhosis, antibacterial drugs, upper gastrointestinal bleeding, portal hypertension, meta-analysis, cephalosporin, quinolone, β-lactams

## Abstract

**Background:** Prophylactic antibacterial drugs are used for patients with liver cirrhosis and upper gastrointestinal bleeding, and independent studies have concluded that they can decrease the rate of infection, mortality, and rebleeding in these diseases. However, no comprehensive assessment of this effect has been reported in recent years and available data pertaining to the prognostic implications of diverse categories of antibiotic prophylaxis in individuals afflicted with cirrhosis are notably limited. The objective of this article is to assess the clinical effectiveness of prophylactic antibacterial drugs for patients with liver cirrhosis and upper gastrointestinal bleeding.

**Methods:** Relevant randomized controlled studies and cohort studies which examined the value of prophylactic antibacterial drugs for patients with liver cirrhosis and upper gastrointestinal bleeding were retrieved via Cochrane Library, EMBASE, MedLine, and Web of Science. The search period was from database inception until 30 April 2023. Summing up the relevant data, the dichotomous variable was statistically analysed using the relative risk (RR) value and its 95% confidence interval (CI) and the continuous variable using the mean difference (MD) value and its 95% CI. All analyses were performed using Revman 5.4 software. The study has been registered on the PROSPERO website under registration number CRD42022343352.

**Results:** Twenty-six studies (18 RCTs and 8 cohort studies, including 13,670 participants) were included to evaluate the effect of antibacterial prophylaxis *versus* no antibacterial prophylaxis or placebo. Prophylactic antibiotics reduced mortality rates (RR 0.66, 95% CI 0.51–0.83), infection rates (RR 0.41, 95% CI 0.35–0.49), rebleeding rates (RR 0.42, 95% CI 0.31–0.56), and length of hospital stay (MD −5.29, 95% CI −7.53, −3.04). Subgroup analysis revealed that the prophylactic administration of quinolone antimicrobials demonstrated the most favorable efficacy, followed by cephalosporins. Both interventions were effective in averting infections frequently observed in patients with liver cirrhosis and upper gastrointestinal bleeding.

**Conclusion:** Based on our investigation, the prophylactic antibacterial drugs confers noteworthy advantages in patients afflicted by liver cirrhosis with upper gastrointestinal bleeding. It has been associated with reductions in mortality, infection incidence, rebleeding occurrences, and the duration of hospitalization. Among prophylactic antibacterial options, quinolones emerged as the foremost choice, with cephalosporins ranking closely thereafter.

Systematic Review Registration: https://www.crd.york.ac.uk/prospero/display_record.php?ID=CRD42022343352, identifier CRD42022343352.

## Introduction

Cirrhosis is a chronic and progressive condition stemming from a diverse array of etiological factors. It is typified by widespread hepatocellular degeneration, necrosis, aberrant hepatocyte proliferation, intrahepatic vascular neovascularization, extensive hepatic fibrotic tissue proliferation, and the development of pseudo-lobar structures (Yoshiji et al., 2021). Cirrhosis is marked by hepatic hypoplasia and the onset of portal hypertension, often leading to complications in the decompensated stage, including esophagogastric variceal hemorrhage, spontaneous bacterial peritonitis, hepatic encephalopathy, hepatorenal syndrome, portal vein thrombosis, and others (Ge and Runyon, 2016; Angeli et al., 2018).

Data from previous studies show that approximately half of patients with liver cirrhosis have upper gastrointestinal varices. Patients with cirrhosis of varying grades exhibit differing risks of developing varices, with 40% of Child-Pugh Class A patients and 85% of Child-Pugh Class C patients developing the condition (Kim et al., 2010). The development of varices can pose a significant risk of upper gastrointestinal bleeding, with a first variceal rupture carrying a mortality rate of 30%–35% (Chinese Society of Spleen and PortalHypertension Surgery et al., 2019), underscoring the gravity of condition. A guideline (Biggins et al., 2021) published by the American Association for the Study of Liver Diseases (AASLD) in 2021 and Baveno VII workshop (de Franchis et al., 2022) recommended short-term antibacterial prophylaxis for any patient with upper gastrointestinal bleeding in liver cirrhosis.

This article encompassed published research on the impact of prophylactic antibacterial drugs for patients afflicted by liver cirrhosis with upper gastrointestinal bleeding. Our analysis includes a comprehensive spectrum of evidence, comprising randomized controlled trials and cohort studies. Separate meta-analyses of these study types were conducted to scrutinize the efficacy of prophylactic antibacterial administration in this patient population. Furthermore, we aimed to discern the most suitable category of antibiotics for employment in patients with this condition, with the overarching goal of optimizing clinical outcomes for these individuals. The study has been registered on the PROSPERO website under registration number CRD42022343352.

## Materials and Methods

### Inclusion criteria

The study’s inclusion criteria adhered to the PICOS framework. Specifically, the participants were patients diagnosed with cirrhosis who experienced upper gastrointestinal bleeding. The intervention examined was the prophylactic administration of antibacterial drugs. The control group comprised patients who did not receive antibacterial drugs or received a placebo. The main outcomes of this meta-analysis were infection rate and mortality rate, whereas secondary outcomes were rebleeding rate and number of hospital stay days. Finally, the study design included both randomized controlled trials (RCTs) and cohort studies.

### Screening studies

RCTs and cohort studies published up to 30 April 2023, from MedLine (via PubMED), China National Knowledge Internet (CNKI) (via Web of Science or Google Scholar), Embase and Cochrane Library databases were searched using computers. The searched keywords included “antibiotic prophylaxis”, “liver cirrhosis”, “gastrointestinal hemorrhage” and their synonyms and combinations. Specific and detailed PubMed search strategies are provided in the Supplementary Appendix.

### Inclusion of studies and data extraction

Two authors, namely, WZ and HHS, conducted an independent review of each identified article and adhered to predefined inclusion criteria for the selection of studies earmarked for subsequent meta-analysis. Both authors utilized a consistent framework to autonomously extract key information from the included studies, encompassing details such as the literature’s authors, publication year, geographical context, study design, sample size, antimicrobial interventions, and study outcomes. In cases of disagreement between these two researchers, a final resolution was reached through the consultation of a third researcher, LXD. All authors engaged in comprehensive discussions and diligently documented the extracted data.

### Quality assessment of the included studies

The Newcastle–Ottawa scale (NOS) (Stang, 2010) was used to assess the quality of the cohort studies, with <6 indicating a low-quality study and ≥6 indicating a high-quality study. For RCTs, we used the Cochrane risk bias assessment tool (Higgins et al., 2011) for quality evaluation.

### Integration and analysis of data

The study was meta-analysed using the RevMan 5.4 software. For the extracted dichotomous variables, we used Relative risk (RR) values and their 95% confidence intervals (CI) for statistical analysis, and for the extracted continuous variables, we used Mean Difference (MD) values and their 95% confidence intervals for statistical analysis.

All included studies were tested for heterogeneity and were considered not to be statistically heterogeneous when *p* > 0.10 or I^2^ <50%. Sensitivity analyses were also performed between studies with heterogeneity to identify the sources of heterogeneity. A fixed-effects model was used for studies without heterogeneity. Otherwise, a random-effects model was used.

## Results

### Study characteristics

**FIGURE 1:**
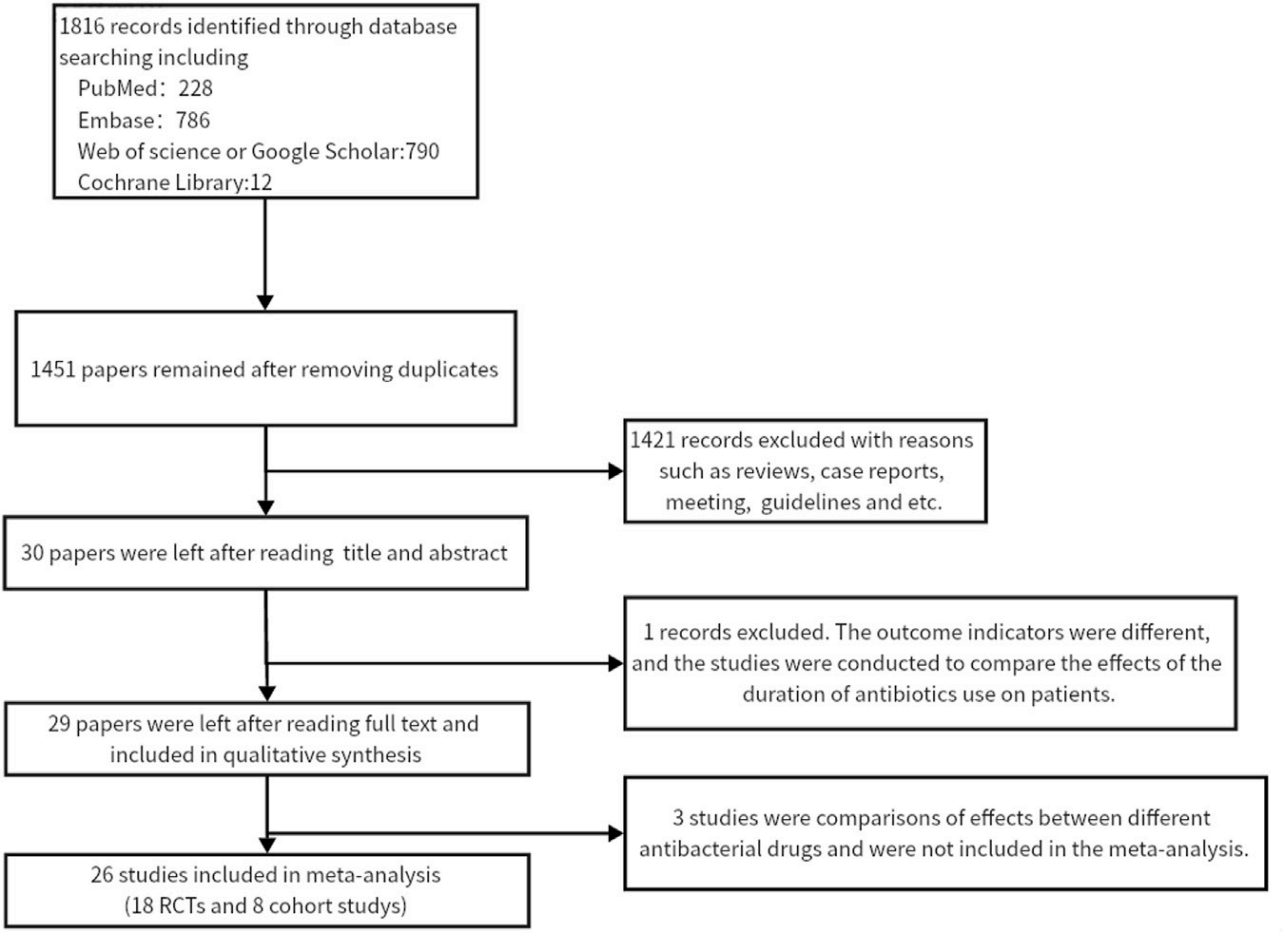
flowchart of the literature search process.

Twenty-six studies (Wu et al., 2020; Chang et al., 2020; Tandon et al., 2015; Xu et al., 2011; Ueno et al., 2020; Fu and Yang, 2015; Moon et al., 2016; Y et al., 2023; Blaise et al., 1994; Soriano et al., 1992; Rimola et al., 1985; Jiang et al., 2018; Jun et al., 2006; Wu et al., 2002; Hsieh et al., 1998; Wu, 2019; Na, 2017; Yang et al., 2015; Yu et al., 2013; Zhu et al., 2011; Hou et al., 2004; Rolando et al., 1993; Selby et al., 1994; Xu, 2021; Qin, 2020; Zhang and Ma, 2018) were included in this meta-analysis (13,670 participants). Figure 1 shows the PRISMA flowchart of the literature search process. Table 1 summarises the specific information of all studies included in the quantitative analysis, which compared the effects of antibiotic prophylaxis using cephalosporins (13 trials) (Selby et al., 1994; Jun et al., 2006; Xu et al., 2011; Zhu et al., 2011; Fu and Yang, 2015; Moon et al., 2016; Na, 2017; Jiang et al., 2018; Zhang and Ma, 2018; Wu, 2019; Chang et al., 2020; Ueno et al., 2020; Xu, 2021), quinolones (4 trials) (Soriano et al., 1992; Blaise et al., 1994; Hsieh et al., 1998; Hou et al., 2004), cephalosporins or quinolones (6 trials) (Wu et al., 2020; Tandon et al., 2015; Yang et al., 2015; Yu et al., 2013; Qin, 2020; Y et al., 2023), quinolones or penicillin (1 trial) (Wu et al., 2002), and other antibiotics (2 trials) (Rimola et al., 1985; Rolando et al., 1993) *versus* no intervention or placebo.

**TABLE 1:** Trials comparing intervention with placebo or no intervention.

Study	Country/Region	Study type	Intervention	Control	Outcome^†^
Blaise (1994)	France	RCT	Intravenous ofloxacin 0.4 g daily for 10 days	No antibiotic prophylaxis	①④
Hou (2004)	Taiwan	RCT	Intravenous ofloxacin 0.2 g b.d. for 2 days and followed by oral ofloxacin 0.2 g b.d. for 5 days	On-demand antibiotics	③④
Hsieh (1998)	Taiwan	RCT	oral or nasal feeding, ciprofloxacin 0.5 g b.d. for 7 days	Placebo	①②③④
Jiang (2018)	China	RCT	Intravenous cefoperazone sodium and sulbactam sodium	No antibiotic prophylaxis	①③④
Jun (2006)	Korea	RCT	Intravenous cefotaxime 2.0 g every 8 h for 7 days	On-demand antibiotics	①③④
Na (2017)	China	RCT	intravenous cefotaxime 2.0 g b.d	No antibiotic prophylaxis	①②③④
Qin (2020)	China	RCT	oral norfloxacin 0.4 g everyday or intravenous ceftriaxone sodium 1.0 g everyday	No antibiotic prophylaxis	①③④
Rimola (1985)	Spain	RCT	First group: oral gentamicin (0.2 g) + vancomycin (0.5 g) + nystatin (10^6^ UI) every 6 h; Second group: neomycin (1 g) + colistin (1.5 × 10^6^ UI) + nystatin (10^6^ UI) every 6 h	No antibiotic prophylaxis	①④
Rolando (1993)	United Kingdom	RCT	Intravenous imipenem/cilastatin	Infuse dextrose-saline	①④
Selby (1994)	Australia	RCT	Intravenous cefotaxime	No antibiotic prophylaxis	①
Soriano (1992)	Spain	RCT	Oral or nasal feeding, norfloxacin 0.2 g, b.d. for 7 days	No antibiotic prophylaxis	①④
Wu (2002)	China	RCT	Intravenous ciprofloxacin 0.2 g b.d. or intravenous piperacillin b.d	No antibiotic prophylaxis	①④
Wu (2019)	China	RCT	Intravenous ceftazidime	No antibiotic prophylaxis	①②③④
Xu (2021)	China	RCT	Intravenous Cefoperazone sulbactam 3.0 g b.d	No antibiotic prophylaxis	①③④
Yang (2015)	China	RCT	Quinolones or the third-generation cephalosporin	No antibiotic prophylaxis	①②③④
Yu (2013)	China	RCT	Intravenous quinolones or ceftriaxone	No antibiotic prophylaxis	①
Zhang (2018)	China	RCT	Intravenous ceftriaxone sodium 1.0 g everyday	No antibiotic prophylaxis	①③④
Zhu (2011)	China	RCT	Ceftazidime	No antibiotic prophylaxis	①②③④
Xu (2011)	Taiwan	cohort	Intravenous cefazolin 1.0 g, every 8 h, for 2–7 days	No antibiotic prophylaxis	①②③④
Fu (2015)	China	cohort	Intravenous cefotaxime 2.0 g b.d	No antibiotic prophylaxis	①②③④
Tandon (2015)	Canada	cohort	Ciprofloxacin, third-generation cephalosporins and other types of antibiotics	No antibiotic prophylaxis	①③④
Moon (2016)	United States	cohort	Fluoroquinolone or third-generation cephalosporin	No antibiotic prophylaxis	④
Wu (2020)	Taiwan	cohort	Cephalosporin or quinolones	No antibiotic prophylaxis	③④
Chang (2020)	Taiwan	cohort	Cefazolin or cefuroxime or ceftriaxone or cefazolin plus gentamicin	No antibiotic prophylaxis	①②③
Uneo (2020)	Japan	cohort	Cefazolin or ceftriaxone	No antibiotic prophylaxis	①③④
B.hadiY 2023	United States	cohort	Ceftriaxone, fluoroquinolones or meropenem	No antibiotic prophylaxis	①

†① = infection rate ② = length of hospital stay ③ = rebleeding rate ④ = mortality rate b.d. is defined as the administration of medication twice daily.

### Quality assessment of studies

**FIGURE 2:**
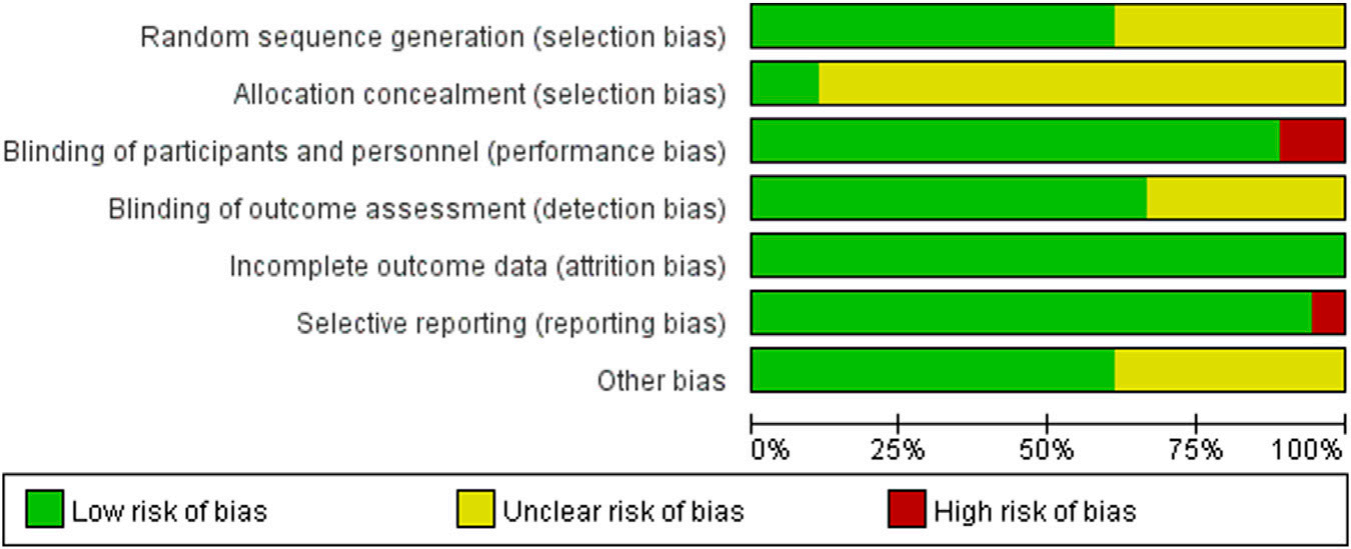
Quality assessment of RCTs.

The 18 RCTs (Rimola et al., 1985; Soriano et al., 1992; Rolando et al., 1993; Blaise et al., 1994; Selby et al., 1994; Hsieh et al., 1998; Wu et al., 2002; Hou et al., 2004; Jun et al., 2006; Zhu et al., 2011; Yu et al., 2013; Yang et al., 2015; Na, 2017; Jiang et al., 2018; Zhang and Ma, 2018; Wu, 2019; Qin, 2020; Xu, 2021) included in this meta-analysis had a partial risk of bias, with lack of blinding or imperfect blinding being the most significant sources of bias. Most of the cohort studies were of high quality. Figure 2 shows the risk bias assessment graph for the RCT and Table 2 shows the quality evaluation of the cohort studies.

**TABLE 2:** Quality evaluation of cohort studies.

Study	Selection				Comparability	Outcome			Quality score
	Representativeness of the exposed cohort	Selection of the non-exposed cohort	Ascertainment of exposure	Demonstration that outcome of interest was not present at the beginning of study	Comparability of cohorts based on the design or analysis	Assessment of outcome	Was follow-up long enough for outcome to occur	Adequacy of follow-up of cohort	
Xu (2011)	1	1	1	0	1	1	0	0	5
Fu (2015)	1	1	1	0	1	1	1	1	7
Tandon (2015)	1	1	1	0	1	1	1	1	7
Moon (2016)	1	1	1	0	1	1	1	1	7
Wu (2020)	1	1	0	0	1	1	1	1	6
Chang (2020)	1	1	1	0	2	1	1	1	8
Ueno (2020)	1	1	1	0	2	1	0	0	6
B. Hadi Y (2023)	1	1	1	0	2	1	0	0	6

### Analysis of the results

#### The rate of mortality

**FIGURE 3:**
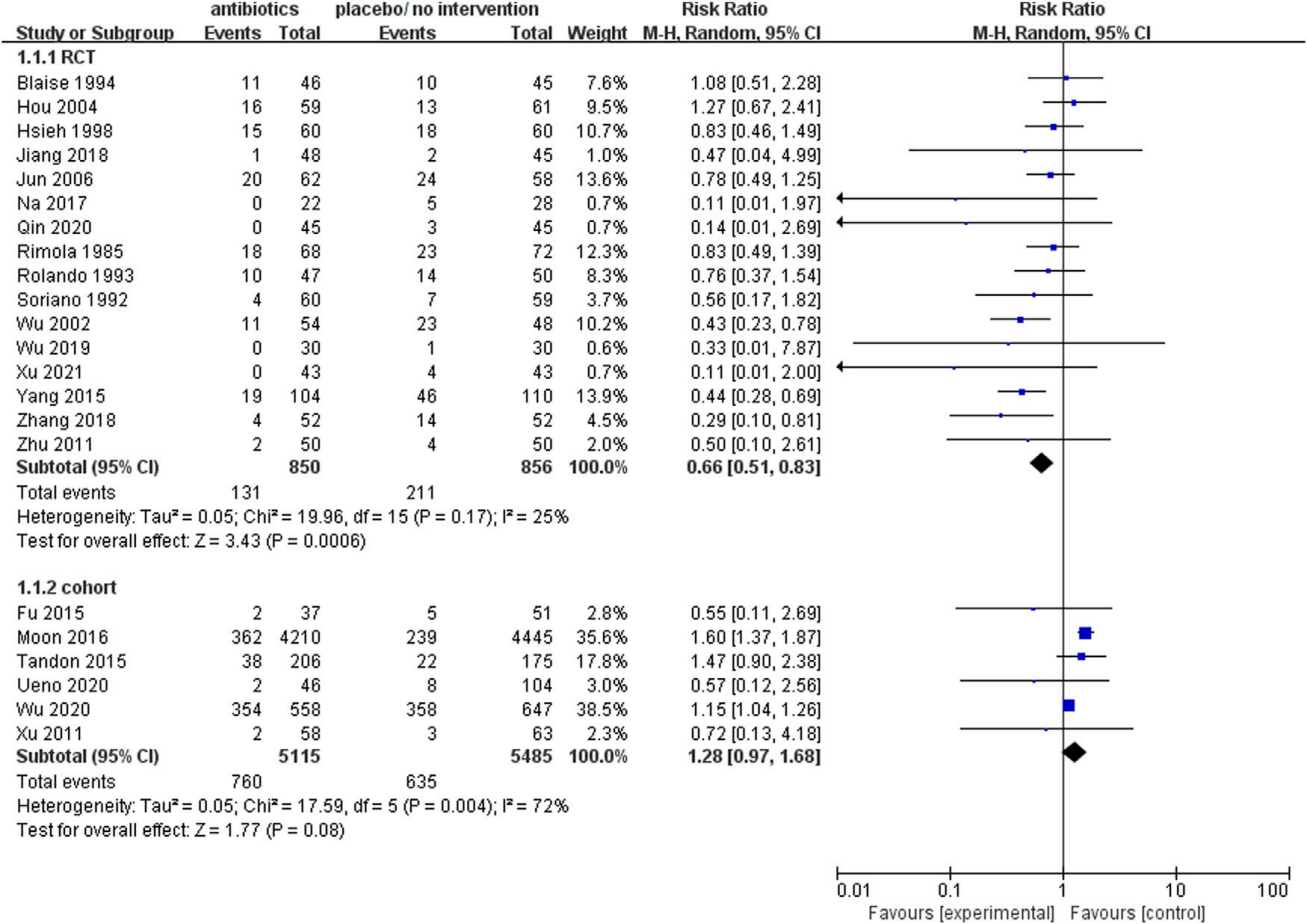
Forest plot: prophylactic antibacterial drugs vs. no prophylaxis/placebo, outcome: mortality rate.

Upon analysis of the incorporated randomized controlled trials, it was observed that the prophylactic antibacterial drugs resulted in a reduction of patient mortality (RR 0.66; 95% CI 0.51–0.83, *p* = 0.0006). Conversely, the cohort study did not yield statistically significant findings (Figure 3). What’s more, the cohort study exhibited substantial heterogeneity in the analysis (I^2^ = 72%), thus necessitating the implementation of a random effects model. The findings present robust evidence in favor of the dependability and feasibility of prophylactic employment of antimicrobial agents. Nevertheless, the inherent challenges in mitigating confounding biases in cohort studies diminish the reliability of their results compared to randomized controlled trials (RCTs). It was observed that, in specific cohort studies (Moon et al., 2016; Wu et al., 2020), the group subjected to antimicrobial prophylaxis exhibited an elevated mortality rate. This discrepancy may be attributed to variations in disease severity among individuals in this cohort, where those receiving antibacterial prophylaxis often presented more advanced disease processes. In contrast, RCTs, with their random allocation methodology, effectively address this issue, thus ensuring a heightened level of validity and dependability in the research outcomes.

#### The rate of infection

**FIGURE 4:**
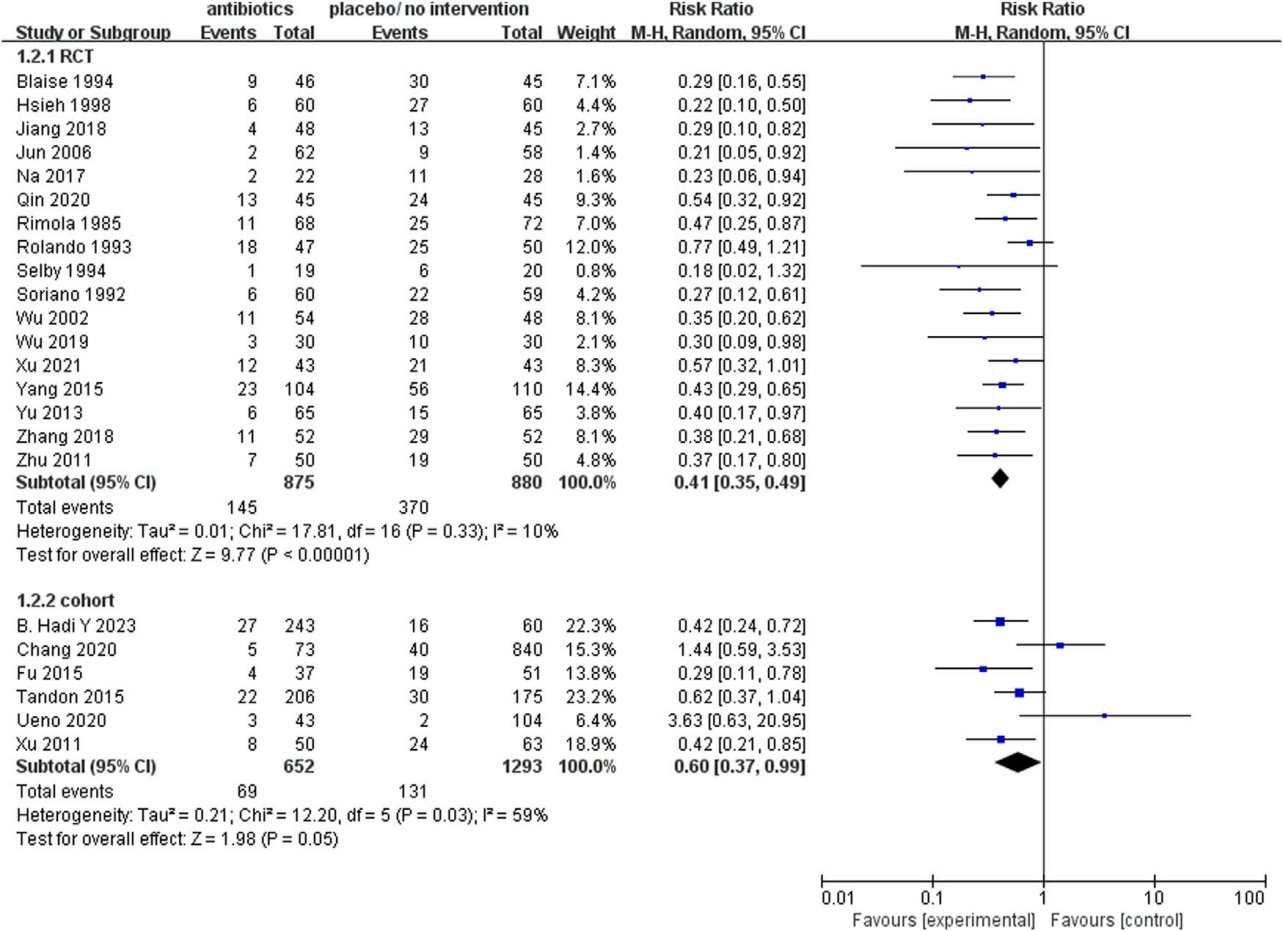
Forest plot: prophylactic antibacterial drugs vs. no prophylaxis/placebo, outcome: infection rate.

The analysis of 17 RCTs, encompassing a total of 1755 patients, revealed that the prophylactic antibacterial drugs was associated with a significant reduction in patient infection rates (Figure 4; RR 0.41; 95%CI 0.35–0.49, *p* < 0.01). Notably, no statistically significant difference was observed in the cohort studies (RR 0.60, 95% CI 0.37–0.99, *p* = 0.05). Both random effects and fixed effects models consistently yielded results supporting the conclusion that the prophylactic use of antimicrobial agents effectively diminishes the incidence of infections in patients with cirrhosis and upper gastrointestinal bleeding.

Moreover, comprehensive subgroup analyses were undertaken to elucidate the following critical aspects: 1. The efficacy of antibacterial drugs in preventing infections at specific anatomical site. 2. The comparative effectiveness of distinct antimicrobial categories in mitigating the incidence of infections among the 13,670 patients enrolled in this study. Infectious complications found in this study are summarised below, and incidence rates were consistent with those of previous studies.

The analysis revealed the prophylactic antibacterial drugs to be efficacious in preventing infections commonly associated with upper gastrointestinal bleeding in cirrhosis (Supplementary Figure S1A: Forest plot. prophylactic antibacterial drugs vs. no prophylaxis or placebo, outcome is infection rate, grouped according to infection site). Notably, it demonstrated significant reductions in the risk of various infections, including Spontaneous Bacterial Peritonitis (SBP) (RR 0.35; 95%CI 0.23–0.54), respiratory infections (RR 0.40, 95% CI 0.25–0.63), urinary infections (RR 0.22, 95% CI 0.11–0.42), gastrointestinal infections (RR 0.41, 95% CI 0.16–1.04), and bacteraemia (RR 0.31, 95% CI 0.18–0.53).

Furthermore, within the scope of the included studies, an enumeration of the pathogenic microorganisms responsible for patients’ infections was conducted. It was observed that nine studies reported bacterial culture results, identifying a total of 144 g-negative strains, predominantly comprising *Escherichia coli and Klebsiella pneumoniae,* 69 g-positive strains, primarily *Staphylococcus* spp. and *Enterococcus* spp., along with five fungal strains.

**FIGURE 5:**
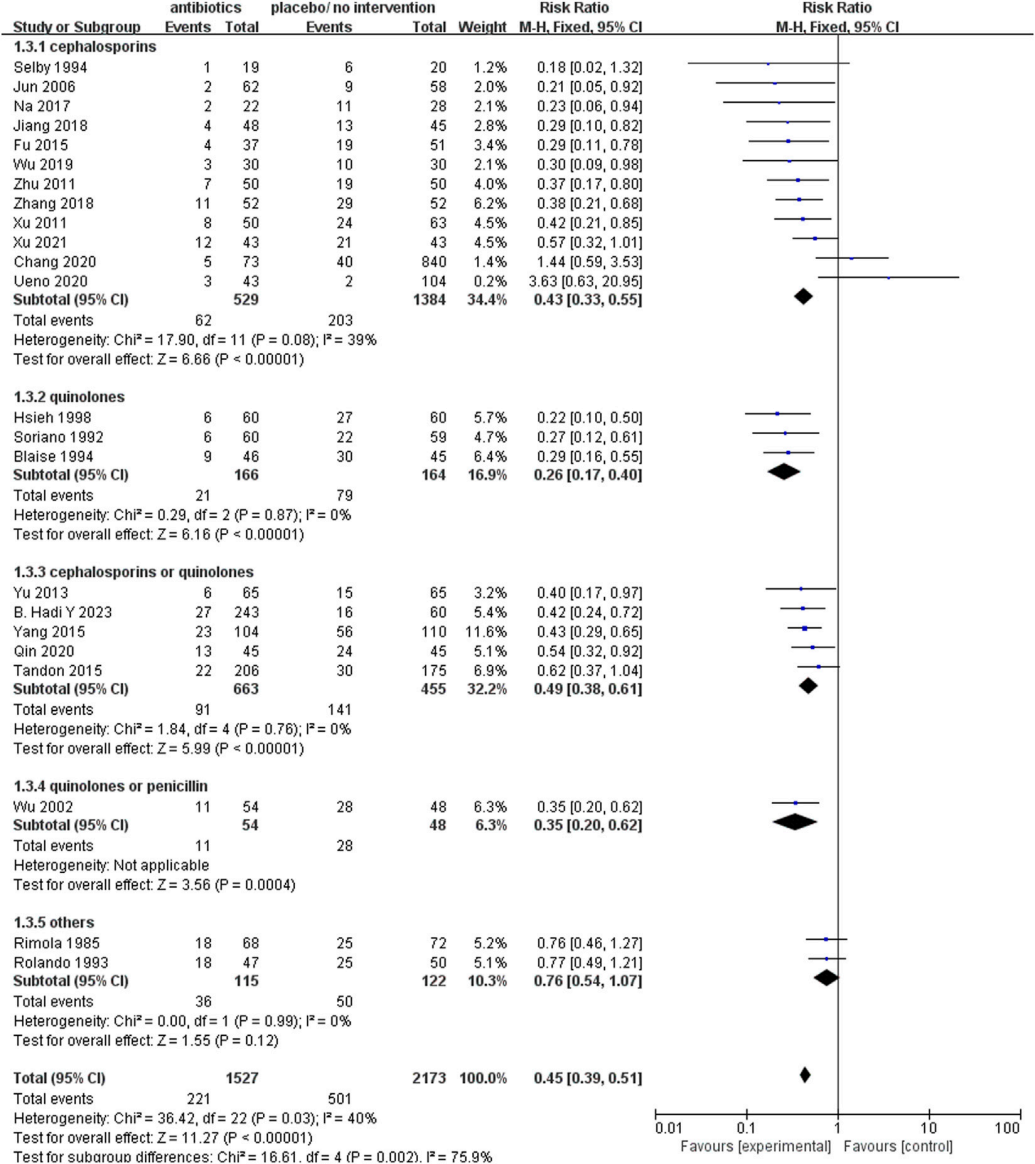
Forest plot: prophylactic antibacterial drugs vs. no prophylaxis/placebo, outcome: infection rate (grouped according to antibacterial drugs).

The prophylactic use of all antibacterial medications encompassed in this analysis yielded a notable reduction in infection rates. The relative risk (RR) values for each class of these drugs are presented in Figure 5, showcasing the following findings: quinolones (RR 0.26; 95% CI 0.17–0.40), quinolones or penicillin (RR 0.35; 95% CI 0.20–0.62), cephalosporins (RR 0.43; 95% CI 0.33–0.55), cephalosporins or quinolones (RR 0.49; 95% CI 0.38–0.61), and other antibacterial drugs (RR 0.76; 95% CI 0.54–1.07).

Furthermore, this analysis showed that all participants who received antibacterial prophylaxis exhibited a significantly reduced rate of rebleeding (RR 0.42; 95% CI 0.31–0.56, *p* < 0.01, Supplementary Figure S1B: Forest plot. prophylactic antibacterial drugs vs. no prophylaxis or placebo, outcome is rebleeding rate) and experienced a shorter hospital stay (MD −5.29; 95% CI −7.53 to −3.04, *p* < 0.01, Supplementary Figure S1C: Forest plot. prophylactic antibacterial drugs vs. no prophylaxis or placebo, outcome is hospital stay). Notably, the analysis of these two outcomes did not reveal statistical significance in the cohort studies.

## Discussion

The primary objective of this meta-analysis was to evaluate the prophylactic use of antimicrobial agents in patients with cirrhosis experiencing upper gastrointestinal bleeding, with a specific focus on demonstrating the substantial associations between prophylactic antimicrobial usage and reduced rates of infections, mortality, rebleeding, and shorter hospital stays.

It is noteworthy that a high-quality systematic review and meta-analysis, akin to the present study, had been previously published in 2011 (Chavez-Tapia et al., 2011). In light of our incorporation of recently published studies, our findings consistently align with the earlier meta-analysis, underscoring the clinical benefits of prophylactic antimicrobial administration in cirrhotic patients with upper gastrointestinal bleeding, where it may effectively mitigate mortality, infection, rebleeding, and duration of hospitalization. Moreover, our study encompasses additional outcome indicators, including the examination of infection types and their distribution, contributing to a more comprehensive understanding of the subject matter.

In a multitude of independent studies, both the relative efficacy of antimicrobial agents when compared to placebos and the comparative effectiveness among different antimicrobial classes were not consistently established as the most optimal choice. Nevertheless, the overarching utilization of antibacterial medications demonstrated clear benefits for patients (Lee et al., 2014), and our subgroup analysis within this study further underscored the commendable effectiveness of the currently employed prophylactic antibacterial agents in clinical practice. Conspicuously, our study posits that quinolone antibiotics and cephalosporin antibiotics may manifest superior preventive capabilities. Nevertheless, the exigency for further pertinent and high-quality clinical research remains palpable to thoroughly assess the risks and assorted patient benefits.

In recent years, the irrational utilization of antibacterial drugs has precipitated the emergence of a myriad of pathogenic bacteria with heightened resistance to a multitude of clinically employed antimicrobial agents, including instances of multidrug resistance (Ardolino et al., 2019), leading to reduce effectiveness of common antibiotics (Piano et al., 2018). The preceding section provides a comprehensive summary of potential complications encountered by patients with cirrhosis and upper gastrointestinal bleeding, encompassing both the incidence and anatomical localization of infections. It is important to note that the gold standard for diagnosing bacterial infections continues to be the cultivation and subsequent culture of microorganisms, despite the ongoing challenges posed by antimicrobial resistance.

Moreover, the Child-Pugh score finds widespread clinical application for the evaluation of patients afflicted by cirrhosis. Among the studies encompassed within this research, it is noteworthy that only some authors delineated patient stratification and subsequently conducted an evaluation of the suitability for prophylactic antimicrobial intervention based on these stratifications. The findings of Chang et al. (Chang et al., 2020) suggest that the utilization of prophylactic antibacterial drugs demonstrated restricted effectiveness in patients categorized under grades A/B. In a similar vein, subsequent to a retrospective study, Tandon et al. (Tandon et al., 2015) also observed that even when patients were classified as grade A and refrained from the use of prophylactic antibacterial drugs, their incidence of infection and mortality remained notably low.

This study is subject to several inherent limitations. Firstly, the blinding protocols in some of randomized controlled trials were notably imperfect. Furthermore, the crucial aspect of allocation concealment was not explicitly reported in any of these included studies, potentially resulting in a diminution of the overall research quality. Of equal significance, it must be emphasized that the individual causes of patient mortality were not systematically categorized within each of the independent studies incorporated into the meta-analysis. Consequently, the absence of such categorization hinders our capacity to conclusively determine whether patient deaths were primarily attributable to diseases or bacterial infections. This complicates the assessment of whether antimicrobial agents directly contribute to patient welfare by reducing mortality or if their primary role lies in infection prevention among elderly patients, thereby indirectly influencing mortality rates. As previously mentioned, the prophylactic antibacterial drugs typically confers benefits for patients in liver cirrhosis with upper gastrointestinal bleeding. Nevertheless, the limited number of studies that have employed stratification based on the Child-Pugh scoring system leaves the matter unresolved as to whether variances exist in the effectiveness of antibacterial drugs within the A, B, and C classifications. The duration of prophylactic antimicrobial use has also not been evaluated in enough studies. Further clinical investigations of superior quality are imperative to elucidate this matter definitively.

In conclusion, our meta-analysis has determined that the clinical prophylactic antibacterial drugs yields favorable results in patients suffering from liver cirrhosis with upper gastrointestinal bleeding. Quinolones exhibit a superior prophylactic effect, followed by cephalosporins. Nevertheless, further high-quality real-world studies are imperative to comprehensively evaluate the optimal duration of antibacterial drugs and the divergent impacts on patients classified as Child-Pugh A,B, or C.

## Data Availability

The original contributions presented in the study are included in the article/Supplementary Material, further inquiries can be directed to the corresponding authors.
