# Supplementary figures and images for: Effectiveness of prophylactic antibacterial drugs for patients with liver cirrhosis and upper gastrointestinal bleeding: a systematic review and meta-analysis
Source: Front Pharmacol. 2024 Mar 14;15:1324848. doi: 10.3389/fphar.2024.1324848 (PMC10973544; doi:10.3389/fphar.2024.1324848)

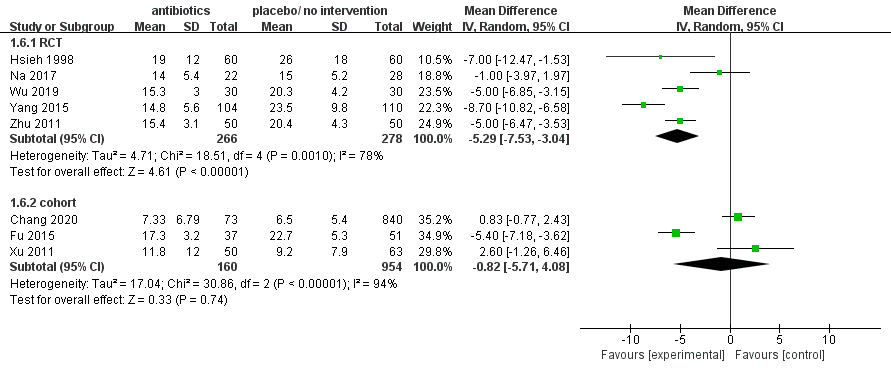

Supplement: Supplementary file 1 [file Image3.TIFF]

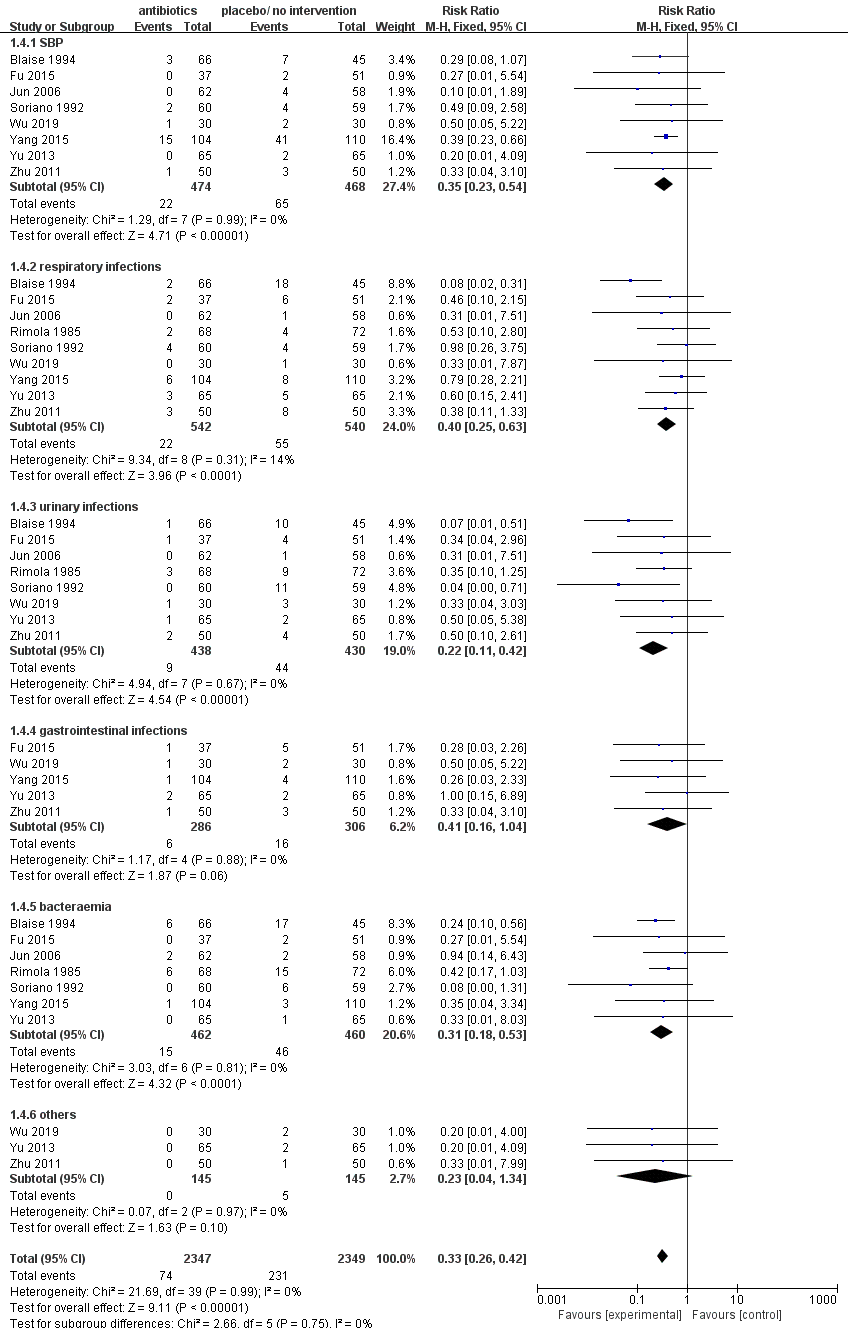

Supplement: Supplementary file 2 [file Image1.TIFF]

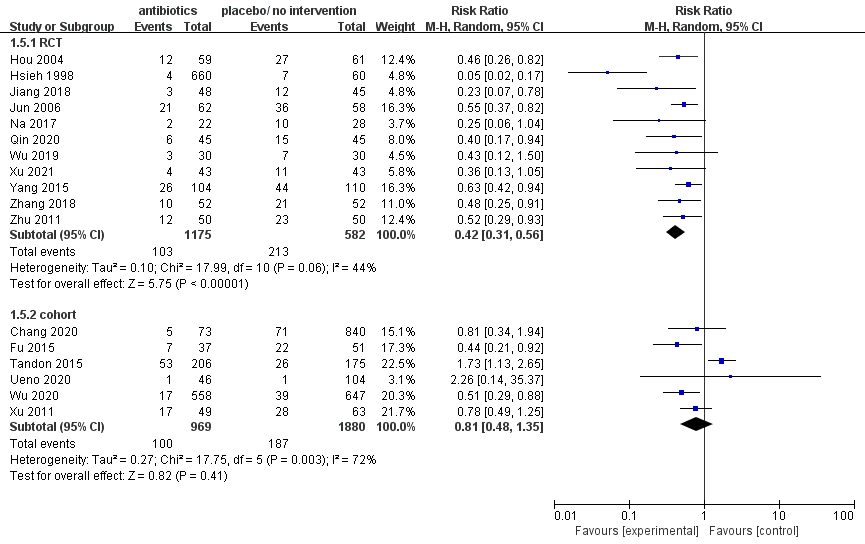

Supplement: Supplementary file 4 [file Image2.TIFF]
