# Supplementary material for: Effectiveness of prophylactic antibacterial drugs for patients with liver cirrhosis and upper gastrointestinal bleeding: a systematic review and meta-analysis
Source: Front Pharmacol. 2024 Mar 14;15:1324848. doi: 10.3389/fphar.2024.1324848 (PMC10973544; doi:10.3389/fphar.2024.1324848)
Supplement: Supplementary file 3 [file DataSheet1.pdf]

MEDLINE via PubMed

#1 explode antibiotic-prophylaxis /All subheadings

#2 antibiotic* prophyl*

#3 antibiotic* pre*

#4 #1 or #2 or #3

#5 explode liver-cirrhosis /All subheadings

#6 liver cirrho*

#7 hepatic cirrho*

#8 liver fibro*

#9 #5 or #6 or #7 or #8

#10 explode gastrointestinal-hemorrhage /All subheadings

#11 gastr* hemorrhage

#12 gastr* haemorrhage

#13 gastr* bleeding

#14 #10 or #11 or #12 or #13

#15 #4 and #9 and #14
